# Magnetic resonance imaging phantoms for quality-control of myocardial T1 and ECV mapping: specific formulation, long-term stability and variation with heart rate and temperature

**DOI:** 10.1186/s12968-016-0275-9

**Published:** 2016-09-22

**Authors:** Vassilios S. Vassiliou, Ee Ling Heng, Peter D. Gatehouse, Jacqueline Donovan, Claire E. Raphael, Shivraman Giri, Sonya V. Babu-Narayan, Michael A. Gatzoulis, Dudley J. Pennell, Sanjay K. Prasad, David N. Firmin

**Affiliations:** 1 NIHR Cardiovascular Biomedical Research Unit, Royal Brompton Hospital, Sydney Street, London, SW3 6NP UK; 2 Imperial College, National Heart and Lung Institute, London, UK; 3 Department of Biochemistry, Royal Brompton Hospital, Sydney Street, London, SW3 6NP UK; 4 Siemens Medical Solutions USA, Inc, Chicago, USA

**Keywords:** T1 mapping, Phantoms, Stability, Agarose, Nickel

## Abstract

**Background:**

Magnetic resonance imaging (MRI) phantoms are routinely used for quality assurance in MRI centres; however their long term stability for verification of myocardial T1/ extracellular volume fraction (ECV) mapping has never been investigated.

**Methods:**

Nickel-chloride agarose gel phantoms were formulated in a reproducible laboratory procedure to mimic blood and myocardial T1 and T2 values, native and late after Gadolinium administration as used in T1/ECV mapping. The phantoms were imaged weekly with an 11 heart beat MOLLI sequence for T1 and long TR spin-echo sequences for T2, in a carefully controlled reproducible manner for 12 months.

**Results:**

There were only small relative changes seen in all the native and post gadolinium T1 values (up to 9.0 % maximal relative change in T1 values) or phantom ECV (up to 8.3 % maximal relative change of ECV, up to 2.2 % maximal absolute change in ECV) during this period. All native and post gadolinium T2 values remained stable over time with <2 % change.

Temperature sensitivity testing showed MOLLI T1 values in the long T1 phantoms increasing by 23.9 ms per degree increase and short T1 phantoms increasing by 0.3 ms per degree increase. There was a small absolute increase in ECV of 0.069 % (~0.22 % relative increase in ECV) per degree increase.

Variation in heart rate testing showed a 0.13 % absolute increase in ECV (~0.45 % relative increase in ECV) per 10 heart rate increase.

**Conclusions:**

These are the first phantoms reported in the literature modeling T1 and T2 values for blood and myocardium specifically for the T1mapping/ECV mapping application, with stability tested rigorously over a 12 month period. This work has significant implications for the utility of such phantoms in improving the accuracy of serial scans for myocardial tissue characterisation by T1 mapping methods and in multicentre work.

## Background

Over the last few decades, cardiovascular magnetic resonance has emerged as an important imaging modality in clinical cardiology and research. However, to ensure accurate results, magnetic resonance imaging (MRI) phantoms have become essential in the development and adjustment of MRI systems as well as maintenance and further evaluation of performance. The recent advent and popularity of MRI based parametric mapping techniques [1] has enabled significant opportunity for enhanced myocardial tissue characterisation. Myocardial T1 mapping methods provide a robust technique [2] frequently used both clinically and in research across a wide spectrum of cardiac conditions including cardiac infiltration [3, 4, 5], inflammation [6, 7], coronary artery disease [8, 9] and siderosis [10]. However, they are well known to be potentially biased [1, 11] to a degree that can depend on protocol parameters, sequence and reconstruction used, with many hidden parameters that are difficult to hold constant over long periods over system upgrades and especially unlikely to be replicated precisely between different models of scanners. Therefore, phantoms have been recommended for Quality Assurance (QA) [11, 12] to provide adequate quality control and protection against scheduled or unplanned changes or failures in the MRI system, control for longitudinal (long-term over time) stability and potentially assist calibration between values obtained from multicentre work. The usefulness of the phantoms lies in their unique ability to provide reassurance against such changes during a research project or in support of clinical applications, and provide basic data that could enable correction across such a change if it were to occur. However phantom-based quality assurance has not been undertaken robustly over a long-term period for T1 mapping sequences.

Previous work on MRI phantoms succeeded in correctly reproducing T1 and T2 values [13, 14, 15], however, their life expectancy was mainly maintained over only a short period of time [16, 17].

The aims of this study were to:

1. Identify whether blood and myocardial T1 and T2 values in the human range before and late-after administration of gadolinium-based contrast agent (“Gadolinium, Gd”) could be reproduced in phantoms, allowing a model for test values into a phantom extracellular volume fraction (ECV) calculation as part of the QA,
2. Identify whether the phantoms could provide sufficient longitudinal stability over a 12 month period of T1 mapping assessment, enabling them to act as a controls against technical changes, planned or unexpected changes/ upgrades in the MRI system, that could impact on T1 and ECV measurements, and
3. Assess the variation of temperature and heart rate on the T1 values measured.

The assessment of variation with temperature and heart rate (HR) could enable investigation of re-calibration requirements both when used in vivo, e.g. when scanning patients at extreme heart rates, but also in vitro when using phantoms for temporal calibration of a single MRI system but also importantly across multicentre work, where confirmation of consistent T1 bias might be required, or if different provide a means to correct data between the centres. Caution should be used however when considering T1 adjustments for variation in temperature in vivo. Although one would expect only small temperature variation in humans due to the body’s own thermoregulatory control; ex vivo work has shown that the relaxivity of Gd-complexes are temperature dependent [18], therefore one would not be able to accurately predict the Gd in vivo response to temperature using work with Nickel based phantoms.

Calibration of T1 values across many centres would also not be an easy task as all the phantoms need to be scanned at the reference centre (centre 1) with one sequence, transferred safely to the new centre (centre 2) for regular scanning with potentially a different sequence and vendor and then returned to centre 1 for repeat scanning to ensure that there was no drift in the phantoms during transportation. Using values from both centres a specific correction algorithm will then need to be developed. This can become even more complex if two or more additional centres are utilising the same set of phantoms. Additionally, for this work, it was necessary to model both T1 and T2 values for each tissue because T2 can cause bias in the estimation of T1 by the single-breath-hold methods used in cardiac MRI, hence the weekly scans included T2 measurements to investigate whether any potential drifts seen in T1 relaxation times occurred as a result of changes in T2 times.

## Methods

### Phantom materials and recipe

Published T1 values were referenced [19] to create phantoms with targeted T1 values at 1.5 T and 37 °C of:

1. native myocardial T1 1000 ms, T2 50 ms;
2. native blood T1 1450 ms, T2 250 ms;
3. post-Gd myocardial T1 500 ms, T2 45 ms; and
4. post-Gd blood T1 380 ms, T2 140 ms.

Nickel-chloride (NiCl_2_) agarose gel phantoms were made by a reproducible laboratory procedure, calculated to model the above T1 and T2 values. Nickel doped gels were chosen to vary proton T1 rates, whilst T2 rates were determined by the concentration of agarose used as the gelling agent [14, 15]. Other paramagnetic ions were avoided because of their known larger temperature dependence than Nickel [14].

NiCl_2_ (Nickel (II) chloride hexahydrate 99.9999 %, ACROS Organics ™) stock solutions at 1 mM, 3 mM and 6 mM were made for accuracy and stored refrigerated in sealed 1 or 2 L flasks. For preparation of these stock solutions large measuring flasks were used with a 1 or 2 L line on the neck. After its initial opening, the appropriate NiCl_2_ weight was transferred onto a plastic boat and was washed into the flask with de-ionised water. This process was achieved rapidly as NiCl_2_.6H_2_O is highly hygroscopic, and once opened the NiCl_2_ hexahydrate (supplied under a dried atmosphere) could not accurately be reused. The flask was topped up with distilled water, with frequent stops and mixing to form the chosen stock for storage. The preparation was performed at room temperature and no heating was necessary as this compound is easily soluble.

**Fig. 1:**
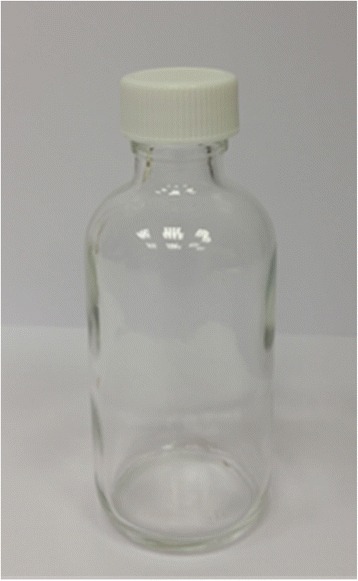
Title: A 60mls narrow neck bottle used. Legend: T﻿he 60mls narrow-neck bottles used (Fisherbrand ^TM^ Clear Soda Lime Glass Boston Round Narrow Mouth Bottles with Polyvinyl Cap) to minimise gel-to-environment exposure by reducing the contact surface area. These were considered more likely to deliver stability than thin-wall tubes with poorly-fitting broad caps

For each required phantom, a conical flask holding 150 ml allowing fast stirring was taken and placed on a chemical balance and its weight was zeroed. The prescribed weight (see below) of agarose (Agarose low EEO for electrophoresis, Mr ≤ 0.07, ACROS Organics ™, nutrient-free) powder was added to the flask. The necessary volumes of the appropriate stock solution and distilled water were added to titrate up to 150 ml total volume. The 150 ml mixture was made as a deliberately large volume to minimise the impact of agar powder weighing and 1 ml fluid measurement errors. For even more accurate results, ideally an even larger mass-production volume could be used. This was achieved by aiming for 1 ml precision of use of the measuring cylinders, using pipettes to assist this process. In this way, agarose, stock solution and distilled water were mixed in specified quantities to derive the selected T1 and T2 values. The specified mixtures were brought near to the boiling-point using a microwave oven until the agarose completely dissolved and a transparent solution was obtained. Excessive boiling was not permitted for fear of losing some of the water content, which would in effect concentrate each mixture inadvertently by an unknown amount. Regular stirring was required to ensure uniformity of the mixture during the agar-dissolution process as the fluid is colourless. In early trial preparations, it was noted that the lack of regular stirring resulted in a non-uniform agarose composition that had to be discarded. The solutions were then carefully transferred into 60 ml glass narrow-neck sealed thick-wall bottles (Fisherbrand ™ Clear Soda Lime Glass Boston Round Narrow Mouth Bottles with Polyvinyl Cap), for minimal vessel permeability and to reduce area of potential contact with air (Fig. 1), filled with minimal gaps resulting from gel contraction while cooling. Two 60 ml bottles were filled from each 150 ml batch. In order to minimise potential B0 distortions in the gel, caution was applied to ensure that any air bubbles were appropriately released from the gel. Failure to do so could have caused the bubbles to become trapped in the solidified gel, leading to increased B0 distortion in the gel around such gaps and making it unacceptable for MRI use; as well as leading to difficulty in drawing regions of interest (ROI) during post-processing to obtain correct T1 values.

Particular care was taken to minimise air entrapment and formation of cracks through cooling of the phantoms as follows:

- Agar sets on cooling at around 45 °C whereas to dissolve it takes at least 95 °C; this hysteresis is well known [20]. On setting, the fluid contracts to form the solid gel. Controlling the setting process in the 60 ml tubes was important to avoid crack formation which could invalidate use of the tubes as MRI phantoms.
- After pouring in the hot gel fluid, the tubes were stood in approximately 1 cm tall cold water bath to cause gel setting from the base upward, so that the contraction of the cooling gel pulled the contents downward, leaving a gap at the top of the phantom.
- Cold water was added to this bath during setting which was visible as white cloudiness in the set gel beginning at the base. This gap was “topped up” with more gel allowed to set until the phantom was filled and the cap screwed on.

As observed from our earlier phases, failure to understand and follow this procedure caused the gel to set beside the vertical walls of the bottle and formation of a large contraction gap down the middle of the bottle rendering it unusable for MRI.

Upon completion of cooling, the phantoms were imaged as described fully later in the methods section, using Siemens 448B Prototype 11 heart beat Modified Look-Locker inversion recovery (MOLLI) for T1 mapping and with long TR (4.5 s) spin-echo sequences for T2 mapping. Multiple iterations of the phantoms were subsequently trialled with adjustments of reagent concentrations, eventually settling on formulations for the targeted T1 and T2 values as tabulated below (Table 1).

**Table 1:** Final relative concentrations of Nickel ion (Ni^2+^) and Agarose required to mimic native and late-Gd human blood and myocardium T1 and T2 values

	Nickel (mM)	Agarose (%)	T1 (ms)	T2 (ms)
Mixture A Native Myocardium	0.90	2.10	1000	55
Mixture B Native Blood	0.48	0.45	1500	225
Mixture C Post-Gd Myocardium	2.35	2.50	510	44
Mixture D Post-Gd Blood	3.70	0.45	365	125

The composition of the 150 ml conical flask contents for these phantoms was obtained by combining:

(A) 0.9 mM Ni^2+^ 2.10 % 3.150 g agarose, 1 mM stock 0.9x150/1 = 135 ml, adding 15 ml water.
(B) 0.48 mM Ni^2+^ 0.45 % 0.675 g agarose, 1 mM stock 0.48x150/1 = 72 ml, adding 78 ml wate.r
(C) 2.35 mM Ni^2+^ 2.50 % 3.750 g agarose, 3 mM stock 2.35x150/3 = 117.5 m, adding 32.5 ml water.
(D) 3.7 mM Ni^2+^ 0.45 % 0.675 g agarose, 6 mM stock 3.7x150/6 = 92.5 ml, adding 57.5 ml water.

The phantoms were observed weekly for evidence of mould growth or cracks in the gel.

### Phantom imaging

The tubes were glued inside a plastic box for protection but no other fill was placed in this outer box. The box contained 8 tubes (two for each mixture A, B, C, D) in a 4x2 grid with 1.5 cm airgaps between the tube outer walls. For imaging, the long axis of each phantom tube was aligned with the z axis of the scanner. Two MRI cylindrical test bottles (diameter 12 cm, height 20 cm, volume 2 L) were placed on each side to support the MRI system (MAGNETOM Avanto, Siemens Healthcare, Erlangen, Germany) calibrations of reference frequency and B1 field, and a transverse slice midway along the 8 tubes within it was imaged following localisers. The adjustment volume, 20 cm x, 12 cm y and 2 cm z, was used to set only the scanner reference frequency because the preset first- and second-order shim settings (known as “Tune-Up” based on a 30 cm-diameter spherical phantom at isocentre) were used without specific adjustment (“shimming”) of these for the T1 mapping phantom.

The phantoms were kept in the same MRI room with a temperature logger, and imaged weekly for 1 year using consistent coil and phantom support arrangements, with the identical sequence version and parameters each week. The image parameters were the same subject to automatic calibrations of flip angle and reference frequency using an identical adjustment volume each week:

1. For T1, MOLLI FOV 360 ×306 mm, slice thickness 8 mm, flip angle 35°, parallel imaging with acceleration factor 2 was used at high-resolution (256 independent pixels over 360 mm and 144 over 306 mm (acquiring 126 by 7/8^th^ partial ky), TR/TE 2.6/1.1 ms, single-shot image acquisition duration 194 ms) at an electronically programmed heart rate of 75 bpm; and low-resolution (192 independent pixels over 360 mm and 128 over 306 mm (acquiring 112 by 7/8^th^ partial ky), TR/TE 2.4/1.0 ms, single-shot image acquisition duration 159 ms) at an electronically programmed heart rate of 100 bpm) versions, with pre-contrast 5(3)3 and post-contrast 4(1)3(1)2 variants. The use of different variations of the MOLLI sequence for the native and post-Gd values has been adopted in line with Schelbert et al. [21] as the two different variations allow better optimisation of images with long and short T1 respectively. This was unlikely to have influenced the longitudinal results as the 5(3)3 sequence was always used for imaging and comparison of the native values and the 4(1)3(1)2 for the post-Gd values.
2. For T2, spin-echo imaging FOV 230 × 108 mm, slice thickness 10 mm, TR/TE 4500/22-264 ms, flip angle 180°.

**Fig. 2:**
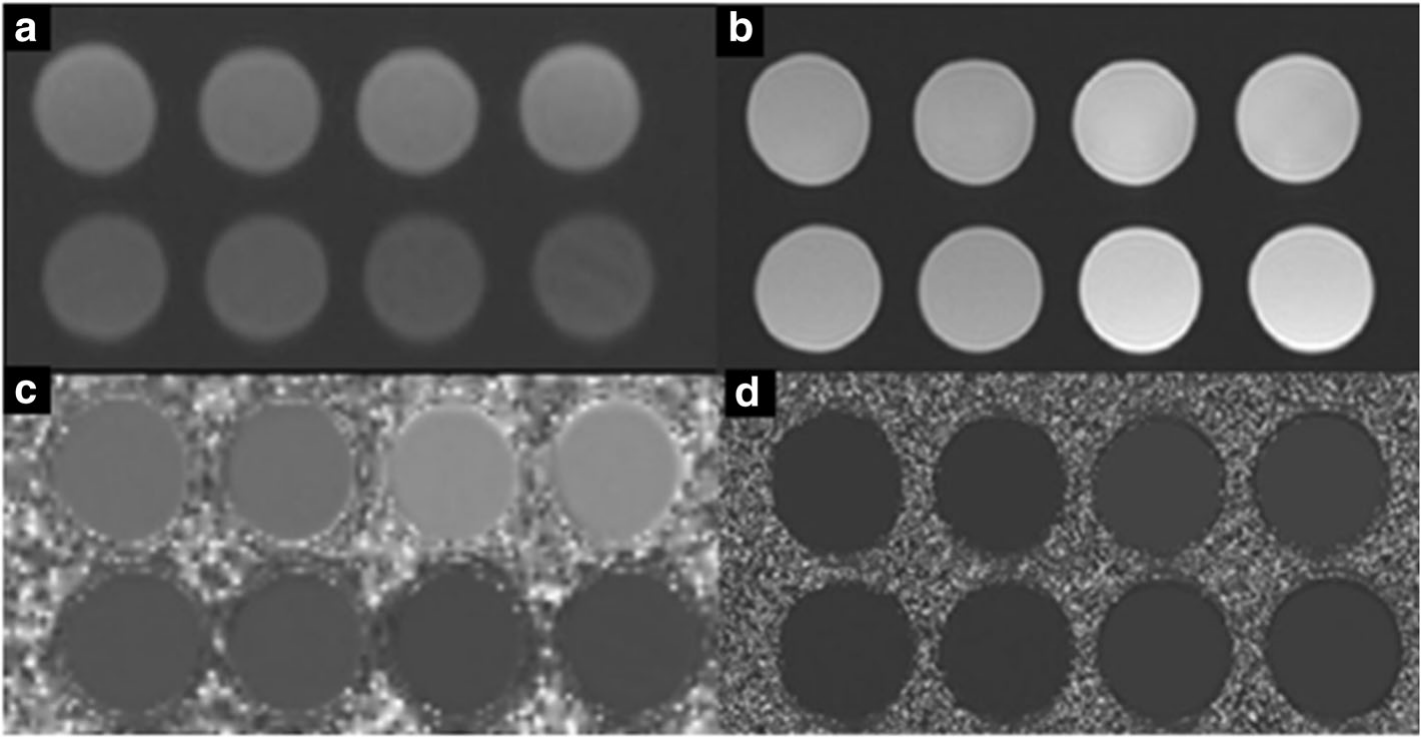
Title: Post processing T1 and T2 images. Each phantom mixture was included twice giving a total of four different mixtures. Legend: Panel **a** is the original inversion recovery image, panel **b** is the spin echo magnitude image. Panels **c** and **d** represent the final maps; panel C is the T1 map and panel D is the T2 map. The ROI mean values were taken well within the phantoms away from edge-ringing artefacts such as Gibbs artefact [26] using CMR Tools

Mean T1 and T2 values were taken in ROIs on pixel-wise maps, examined for drift and analysed on a dedicated software package (CMR Tools, Cardiovascular imaging solutions, London, UK; Fig. 2). The coefficient of variation (CoV = 100× standard deviation/mean) % of 52 weeks was compared against 10 re-positioned repeats acquired within 2 h.

Using the individual components for native and post-Gd blood and myocardium and assuming a “phantom haematocrit” of 0.425 we could calculate a test “extracellular volume fraction” (ECV) of the phantom for assessment of drift impact using the equation:

$$ ECV=\left(1- haematocrit\right)\;\left(\frac{1}{T1\kern0.24em myo\; post}-\frac{1}{T1\kern0.24em myo\;pre}\right)\hbox{---} \hbox{---} \hbox{---} \hbox{---} \hbox{---} \hbox{---} \hbox{---} \hbox{---} \hbox{---} \hbox{---} \hbox{---} \hbox{---} \hbox{---} \hbox{---} \hbox{---} \hbox{---} \hbox{---} \hbox{---} \left(\frac{1}{T1\kern0.24em  blood\; post}-\frac{1}{T1\kern0.24em  blood\;pre}\right) $$

The temperature was measured continuously during the first 6 months using an MR-safe digital thermometer placed on top of the phantom box (during storage, not scanning) to the nearest 0.5 °C. For the remaining 6 months manual read-outs were made from a liquid crystal thermometer strip glued on top of the phantom box at each weekly time scan to the closest 0.5 °C and recorded. The T1 results are reported first as measured and second with application of a temperature-correction to take into consideration the small temperature variation each week. The temperature correction parameters are based on the results of the temperature experiments described below.

There is no standardised method of reporting changes in phantom values, so we calculated the overall change in phantom T1 in two ways, ensuring that we did not underestimate the effect: Firstly, the phantoms were scanned weekly for a year obtaining 52 values per phantom. Four successive weekly values per phantom were averaged resulting in 13 independent values per phantom. The difference between the highest and the lowest value divided by the highest value was calculated as the percentage change. Secondly, the first 4 values and last 4 values were averaged and their difference was divided by the highest value.

### Variation in temperature assessment

**Fig. 3:**
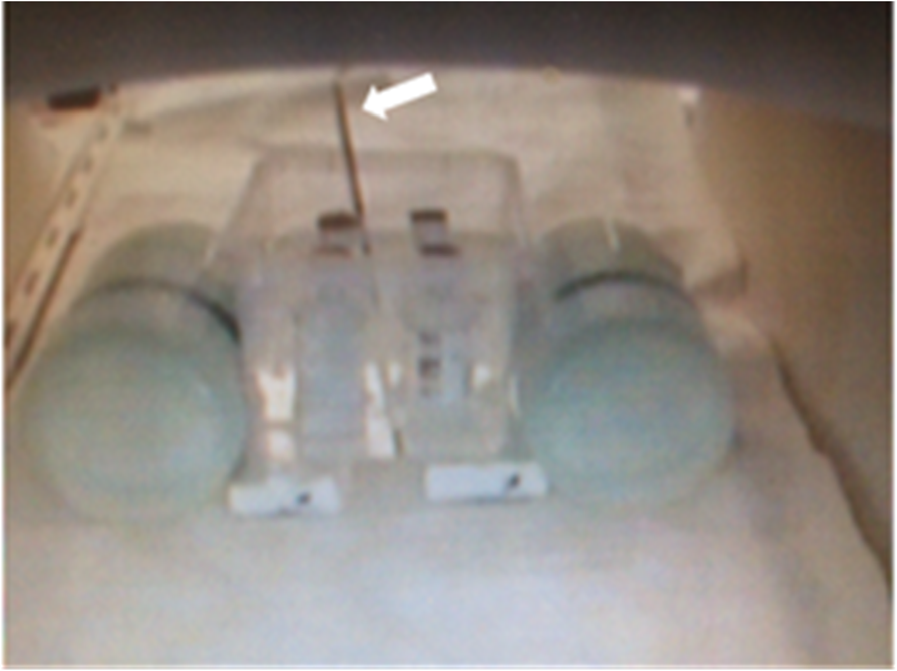
Title: The phantom arrangement during the water bath. Legend: Image showing the phantoms in the water bath, the mercury thermometer (arrow) stabilised in the water bath and the preparation inside the MRI scanner

The effect of variation in temperature on T1 was examined using four separate phantoms with T1 values at 25 °C of 1915, 1040, 530, 280 ms broadly similar to native blood, native myocardium, post-Gd myocardium and post-Gd blood values respectively. A water bath was prepared and the phantoms were immersed and the temperature was manually read using a mercury thermometer to the nearest 0.2 °C (Fig. 3). We started at a temperature of 35 °C cooling down using cold water and finely crushed ice to 15 °C, ensuring careful mixing of the water even after complete melting of the ice to allow a homogeneous temperature whilst imaging at steady HR with real-time reporting of the T1 values. The phantoms used for the temperature experiment were not used in any further longitudinal follow up to avoid any long lasting effect of extreme temperatures on the gels. Of note is the orientation of these phantoms vertically across B0 would not normally be used in air due to B0 distortion effects, but the fluid fill in the box smoothed these out within the central region occupied by the four tubes. This orientation differed from the setup described above for the weekly drift-assessment scans, although the two side bottles were still placed next to the outer box.

### Variation in heart rate assessment

The effect of variation in HR on T1 was also assessed using phantoms with the same values as in Table 1. They were imaged at low resolution (HR > 90 bpm) and high resolution (<90 bpm) for RR 1400-490 ms representing HR 43–122 bpm and at steady temperature, but the initial HR scan was repeated at the end of each series to guard against any unforeseen drift effects.

## Results

### Longitudinal stability of phantom T1 without temperature correction

Both high resolution and low resolution sequences gave similar T1 means, standard deviation and coefficient of variation (CoV) for blood and myocardium native and post-gadolinium (Table 2).

**Table 2:** Showing the variation during a 52 week period of the Phantom unadjusted T1 and T2 values and ECV using an 11-cycle 8-image MOLLI native (pre-Gd) 5(3)3 and post-Gd 4(1)3(1)2. Two phantoms for each T1 and T2 mixture were installed in the box. The value used for analysis represents the average of the two. The phantom ECV was calculated from the myocardial and blood native and post-Gd phantom T1 values using a haematocrit of 0.425. This was a chosen value representing a typical adult human haematocrit

	T1						T2		
	Mean (ms)	SD	CoV (%)	Mean (ms)	SD	CoV (%)	Mean (ms)	SD	CoV (%)
	High resolution (75 bpm)			Low resolution (100 bpm)			Spin-echo protocol		
Native myocardium	971.8	12.6	1.3	971.8	13.6	1.4	56	1.3	2.2
Native blood	1578.8	56.4	3.6	1549.8	61.9	4.0	234	8.0	3.4
Post Gd myocardium	498.3	5.1	1.0	497.6	13.5	1.4	48	0.9	1.9
Post Gd blood	371.9	6.4	1.7	371.8	6.5	1.8	149	3.0	2.0
ECV	27.4 %	0.9	3.2	27.6 %	0.9	3.1			

**Fig. 4:**
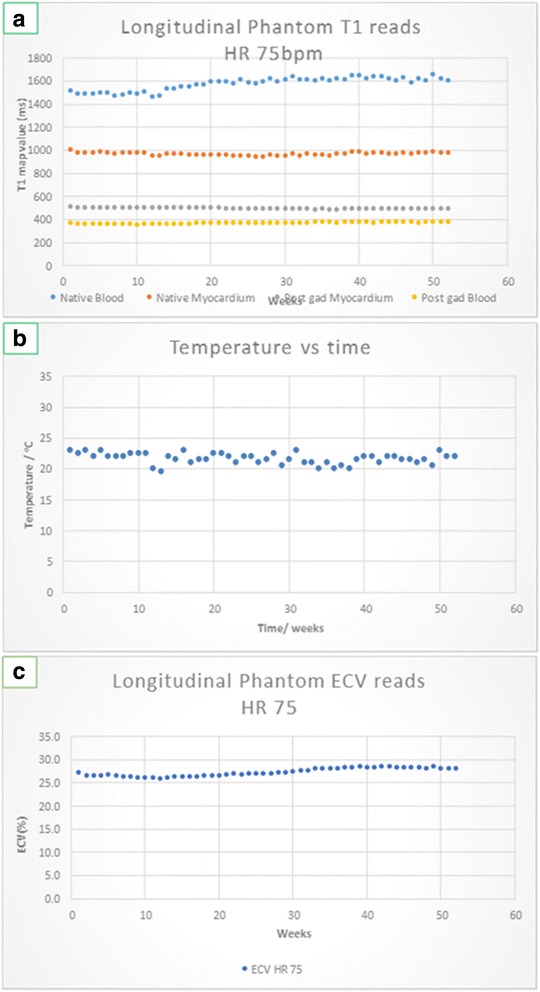
Title: Longitudinal imaging for T1 values and variation in temperature. Legend: Panel **a** longitudinal stability without temperature correction for native and post contrast myocardial and blood phantom T1 values. The phantoms corresponding to native blood T1 values showed a slow drift, whist the remaining phantoms corresponding to native myocardium and post-contrast blood and myocardium remained relatively stable. Panel **b** weekly temperature variation of the MRI room. Panel **c** fusing the individual variables from panel **a** , and a haematocrit of 0.425 a Phantom ECV was calculated

The CoV was higher for the longer T1 and longer T2 values. Specifically for the T1 values, there was no significant drift seen in the shorter T1 values (native myocardium and post-gad blood and myocardium) but there was an increase in the longer native blood value over the 1 year as shown in Table 3 and Fig. 4.

**Table 3:** Showing the overall relative changes in the various T1 parameters over a 12 month period. Independently of method used, all the parameters showed a change of <10 %

	Method 1 (%)	Method 2 (%)
Native blood / ms	9.0	7.8
Native myocardium / ms	3.6	0.5
Post Gd myocardium / ms	2.9	1.9
Post Gd blood / ms	5.0	3.5
ECV / %	8.3	5.3

### Longitudinal stability of phantom T1 with temperature correction

**Fig. 5:**
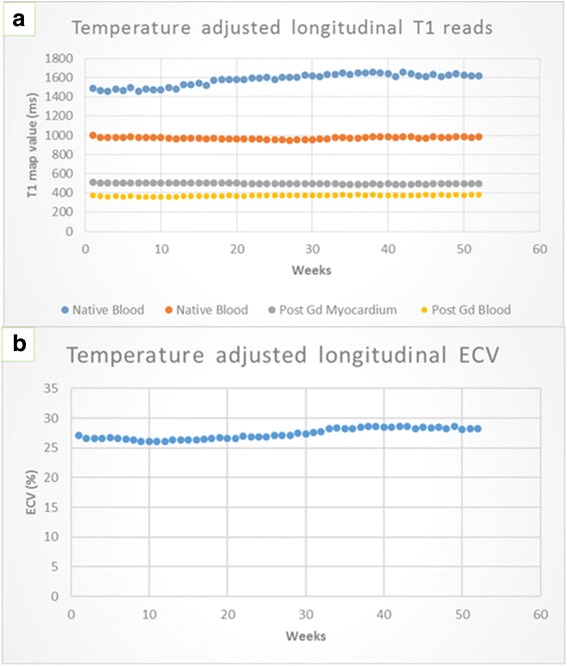
Title: Temperature adjusted T1 values. Legend: Longitudinal follow-up showing adjusted values for T1 (panel **a**) and ECV (panel **b**) incorporating the small temperature variation. The overall results however, of a small drift in the native blood T1 phantoms remained unchanged

When adjusting for the small temperature variations (temperature mean = 21.6 ± 0.9 °C, range 19.5–23 °C) using results from the temperature variation part of the experiment (see below) the results obtained are shown in Fig. 5.

### Long-term temporal variation of T1

The results of phantom T1 change over 12 months are shown in Table 3. Although not the aim of this work, all T2 parameters remained stable with relative <2 % over the 12 month period.

### Effect of temperature change on T1 values

The phantoms were also used to determine the effect of temperature on T1 values. With increase in temperature the very long (similar to pre-Gd blood) and long T1 (similar to pre-Gd myocardium) appeared to increase:

$$ \mathrm{Tube}\ \mathrm{A}\ \left(\mathrm{Native}\ \mathrm{blood}\right) = 23.86 \times \mathrm{Temp} + 1323.3\ \mathrm{ms},{\mathrm{R}}^2 = 0.99,\ \mathrm{p} < 0.0001\ \mathrm{and} $$

$$ \mathrm{Tube}\ \mathrm{B}\ \left(\mathrm{Native}\ \mathrm{Myocardium}\right) = 6.27 \times \mathrm{Temp} + 883\ \mathrm{ms},{\mathrm{R}}^2 = 0.98,\ \mathrm{p} < 0.0001. $$

The shorter T1 values similar to post-Gd myocardium and blood showed only a small association with temperature changes. These followed the equations:

$$ \mathrm{Tube}\ \mathrm{C}\ \left(\mathrm{post}-\mathrm{G}\mathrm{d}\ \mathrm{myocardium}\right) = 0.27\ \mathrm{X}\ \mathrm{Temp} + 525\ \mathrm{ms},\ {\mathrm{R}}^2 = 0.24,\ \mathrm{p} = 0.02, $$

and the

$$ \mathrm{Tube}\ \mathrm{D}\ \left(\mathrm{post}-\mathrm{G}\mathrm{d}\ \mathrm{blood}\right) = 0.81 \times \mathrm{Temp} + 304\ \mathrm{ms},\ {\mathrm{R}}^2 = 0.71,\ \mathrm{p} < 0.001\Big) $$

as shown in Fig. 6.

**Fig. 6:**
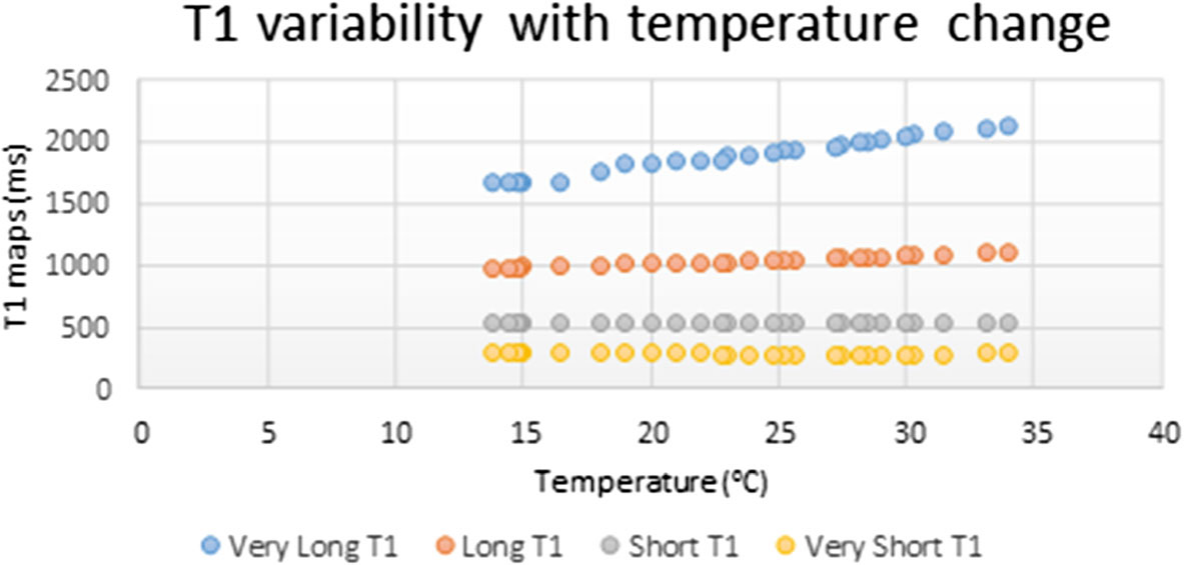
Title: T1 variation with temperature. Legend: Effect of temperature variation on native and post-Gd myocardial and blood T1. This effect appears to predominantly affect the longer T1 values

Putting these changes in the ECV calculator we can see that the increase in ECV would be in the region of 0.1–0.3 ECV units for each degree increase.

An example of incorporating these changes in the ECV calculator is shown below, we can identify the change in ECV per each one degree C difference in temperature.

$$ ECV=\left(1- haematocrit\right)\;\left(\frac{1}{T1\kern0.24em myo\; post}-\frac{1}{T1\kern0.24em myo\;pre}\right)\hbox{---} \hbox{---} \hbox{---} \hbox{---} \hbox{---} \hbox{---} \hbox{---} \hbox{---} \hbox{---} \hbox{---} \hbox{---} \hbox{---} \hbox{---} \hbox{---} \hbox{---} \hbox{---} \hbox{---} \hbox{---} \left(\frac{1}{T1\kern0.24em  blood\; post}-\frac{1}{T1\kern0.24em  blood\;pre}\right) $$

For example assuming that the following values were obtained at temperature X°C, the ECV would correspond to:

$$ ECV\; at\; temperature\;X\circ C=\left(1-0.425\right)\ast \left(\frac{1}{500}-\frac{1}{1000}\right)\hbox{---} \hbox{---} \hbox{---} \hbox{---} \hbox{---} \hbox{---} \hbox{---} \hbox{---} \left(\frac{1}{400}-\frac{1}{1500}\right) $$

Giving an ECV at X°C = 31.36 %.

However, at X + 1 °C using our average change in values for native and post-Gd blood and myocardium the following changes would be expected:

Native blood +23.86 ms, native myocardium +6.27 ms, post-Gd myocardium +0.27 ms, post-Gd blood +0.81 ms.

Putting these new values in the ECV calculator we can calculate the following ECV for temperature X + 1 °C:

$$ ECV\; at\; temperature\;X\circ C=\left(1-0.425\right)\ast \left(\frac{1}{500.27}-\frac{1}{1006.27}\right)\hbox{---} \hbox{---} \hbox{---} \hbox{---} \hbox{---} \hbox{---} \hbox{---} \hbox{---} \hbox{---} \hbox{---} \hbox{---} \left(\frac{1}{400.81}-\frac{1}{1523.86}\right) $$

Giving an ECV at temperature X + 1 = 31.43 %.

This represents an absolute change in ECV of 0.069 % or a relative change in ECV of ~0.22 % per one degree C change in temperature.

### Effect of heart rate variation on T1 values

**Fig. 7:**
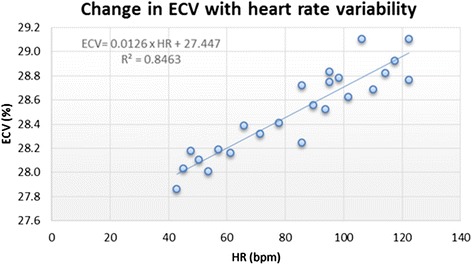
Title: ECV change with heart rate variation. Legend: Graph demonstrating the linear relationship of between HR change and change in ECV. This change was overall small with a 0.13 % absolute increase per 10 heart beat increase

Using the phantoms prepared, the effect of variation in HR was also assessed. There was a small but definite increase in ECV with increase in HR as shown in Fig. 7. This followed a linear relationship:

$$ \mathrm{E}\mathrm{C}\mathrm{V}=0.013 \times \mathrm{H}\mathrm{R} + 28,\ \mathrm{R}2 = 0.85,\ \mathrm{p}<0.0001 $$

Using the phantoms prepared, the effect of HR variation was also assessed. There was a small but definite increase in ECV with increase in HR as shown in Fig. 7. This followed a linear relationship:

$$ \mathrm{E}\mathrm{C}\mathrm{V}=0.013 \ast \mathrm{H}\mathrm{R} + 28,\ \mathrm{R}2 = 0.85,\ \mathrm{p}<0.0001 $$

For example at 50 bpm the ECV is expected to be:

$$ \mathrm{E}\mathrm{C}\mathrm{V}\ \mathrm{at}\ 50\mathrm{bpm} = 0.013 \ast 50 + 28 = 28.65\ \% $$

Whilst at 60 bpm it is expected to be:

$$ \mathrm{E}\mathrm{C}\mathrm{V}\ \mathrm{at}\ 60\mathrm{bpm} = 0.013\ *\ 60 + 28 = 28.78\ \% $$

suggesting a small absolute increase in ECV of 0.13 % for every 10 bpm increase in heart rate, relative increase in ECV of ~0.45 %.

**Fig. 8:**
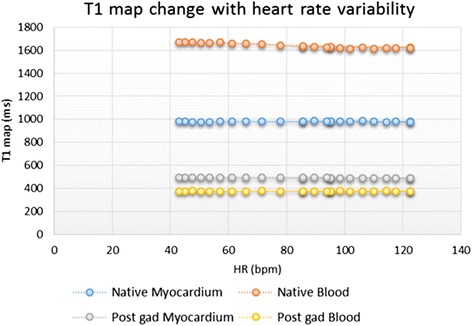
Title: Change in T1 values with heart rate variation. Legend: The effect of heart rate variation in phantoms modeling native and post-Gd myocardium and blood

However, this change in ECV appears to be driven predominantly by a change in native blood T1 (Native blood T1 = −0.78 X HR + 1700, R^2^ = 0.90 *p* < 0.0001) as shown in Fig. 8. As such, if patients with extreme heart rates need to be scanned, native myocardial T1 (Fig. 8), might be more accurate as this showed no significant variation with changes in HR (T1 native myocardium = 0.017 × HR + 981, *R*^2^ = 0.02, *p* = 0.499).

The change in ECV observed with varying heartrate was small; however it appears to relate mainly to a change seen in the native blood values. The remaining values modeling native myocardium, post-Gd blood and post-Gd myocardium appear less sensitive to changes in HR.

## Discussion

### Longitudinal stability of phantoms

We have observed that the change in T1 values observed over a 12 month period was small. This would support that phantoms based on the recipe and construction process we have described could be utilised to provide quality assurance for at least 12 months.

However, this conclusion depends on the exact application of T1 or ECV mapping being monitored. The native myocardial phantom appeared stable whereas the relatively large change in native blood phantom rendered the phantom ECV change from 27.0 to 28.5 % during the year, making phantoms potentially less useful for applications such where ECV is used for assessment of possible subclinical early and small changes in diffuse fibrosis for example. Appreciating that a difference of 3 % in absolute ECV values is associated with significantly worse short-term prognosis [22, 23] and contextualising the drift noticed, we would argue that it is advisable to undertake regular scanning of the phantoms (at least monthly, based on our results) in all units undertaking T1 mapping rather than only opportunistic scanning before any major upgrades, as such an approach could incorrectly estimate the true adjustment required to the ECV values.

Discrepancies arise over the longevity of these agar-based phantoms. Contamination and mould growth, unlike other gels containing nutrients was not seen to occur in any of approximately 100 tubes made on different occasions during this work using nutrient-free agarose, and it is also possible the Ni^2+^ ions also contribute to growth prevention [24], even though complete sterile conditions were not used for manufacture. The instability of gels even if sealed properly inside a tube arises from the contraction of the gel as it dries out. It can do so even within a sealed tube, depositing the water as droplets on the inside of the tube, in the gap left by the contracting gel. The rate at which this occurs is quite uncertain depending on tube and cap design which may allow some water to dry out of the tube, but the effect has still often been noted in well-sealed tubes.

### Variation in temperature and phantom T1

Variation in temperature produced only small changes on the T1 values modeling close to the native and post-Gd myocardium and blood. However, the largest difference was seen on the T1 closer to native blood showing a change of almost 24 ms per 1 °C variation. Importantly, the variation in temperature required to make any of these small changes clinically significant will be very large and is outside the range of temperature changes expected to be seen in clinical practice in the heart.

 Nonetheless, this confirms that for quality assurance across different vendors and sites the phantoms should ideally be imaged in a temperature-controlled environment with minimal changes (<5 °C) and if this is not possible to record and compensate the values accordingly using a correction equation derived from temperature data on the phantoms. Likewise, for comparison of multicentre work in the absence of a correction equation, statistical comparison could be undertaken by employing for example regression analysis using a mixed effects model to assess the impact of systematic inter-centre differences.

### Variation in heart rate and phantom T1

Furthermore, using the phantoms prepared we investigated the effect of HR variation on T1 values. We identified that ECV change was small, estimated at 0.13 ECV units for every increase in 10 bpm. As this difference is small, it suggests that it is unlikely to be relevant for patients in clinical practice unless imaged in decompensated states (e.g. uncontrolled heart failure, fast atrial fibrillation). In such a situation, native myocardial T1 might be a more appropriate parameter to use as it was the most stable with variation in HR, with every 10 bpm increase changing the T1 by 0.17 ms and therefore, might be a more reliable parameter to use when imaging patients at extreme heart rates. This work also supports, that for quality assurance the same HR should be used for longitudinal assessment and also for calibration purposes across various vendors, sites and T1 mapping sequences.

### Study limitations

Firstly, this is a single centre study where phantoms were prepared and imaged in the same institution, therefore did not have to undergo any transport which could have altered their parameters and long term survival. As such, the true usefulness of MRI phantoms in multicenter work can only be assessed once tested in the various centres. Furthermore, the phantoms were prepared in a clinically licensed biochemistry laboratory under standard and reproducible conditions, but sterile conditions were not used. Nonetheless, at 12 months there was no evidence of mould growth or inaccuracies relating to contamination. Neither accuracy data nor magnetisation transfer was assessed, as this experiment focused on long-term precision. Finally, part of the variation noted when HR was tested is because the time allowed for recovery in MOLLI protocols used is applied as counted heartbeats. The duration of this recovery gap therefore varies with HR, likely introducing more error for tubes with longer T1 values that do not recover fully in this time. A modification to MOLLI has been proposed which enforces this gap as a minimum number of seconds not in counted heartbeats [25]. The possible impact of this modified method on the small change seen in the longest T1 tube was not tested in this study.

## Conclusion

We have shown for the first time that Nickel-based phantoms can serve for quality-control over a 12 month period with small changes in native and post-Gd myocardial and blood values and ECV as assessed by an 11 heart beat MOLLI. However, their utility probably requires at least monthly rescanning against phantom drift, to provide some index for correction in the event of a scanner upgrade or modification requiring this.

These results should encourage routine use to support both clinical and research activities using MOLLI sequences.

## Abbreviations

CoV: Coefficient of variation
ECV: Extracellular volume fraction
Gd: Gadolinium
HR: Heart rate
MOLLI: Modified look-locker inversion recovery
MRI: Magnetic resonance imaging
NiCl_2_: Nickel-chloride
QA: Quality assurance
ROI: Region of interest
